# Computation of Intersegmental Moments during Standing Posture: Can We Neglect the Horizontal Ground Reaction Force? Results from an Experimental Study

**DOI:** 10.1155/2019/7129682

**Published:** 2019-11-13

**Authors:** Solène Prost, Sébastien Pesenti, Bertrand Moal, Vincent Pomero, Stephane Fuentes, Patrick Tropiano, Virginie Lafage, Jean-Luc Jouve, Benjamin Blondel

**Affiliations:** ^1^Spine Division, Timone, Aix-Marseille University, 13005 Marseille, France; ^2^Aix Marseille University, Institute of Movement Sciences, CNRS UMR, 7287 Marseille, France; ^3^Pediatric Orthopedics, Timone Children Hospital, Aix-Marseille University, 264 Rue Saint Pierre, 13005 Marseille, France; ^4^Bordeaux University, 146 Rue Léo Saignat, 33000 Bordeaux, France; ^5^Gait Analysis Laboratory, Timone, Aix-Marseille University, 13005 Marseille, France; ^6^Spine Service, Hospital for Special Surgery, New York, NY, USA

## Abstract

**Background:**

The development of postural analysis thanks to force and pressure platforms, in order to determine the center of pressure, can be valuable in the setting of spinal malalignment. The purpose of this study was to compare “pressure” and “force” platforms for the evaluation of the center of pressure. In other words, can we neglect the horizontal ground reaction force in the evaluation of intersegmental moments during standing posture? *Methods*. Postural data from two healthy adult volunteers were analyzed. Analysis of the posture was done according to a protocol providing sagittal intersegmental moments. A set of 36 markers was used to divide the body in 10 segments. Postacquisition calculations were done in order to obtain the sagittal net intersegmental moments. To evaluate the differences in intersegmental moments between force and pressure platforms, the postacquisition calculations were done with a simulated pressure platform. Mean intersegmental moments between each body segment for each volunteer were compared.

**Findings:**

There were significant differences between the 2 platforms in intersegmental moments for the lumbo-sacral junction, hips, knees, and ankles (*p* < 0.005). All differences were inferior to intrasubject variability measured with the force platform (*p* < 0.001). Results from intra- and interobserver comparisons showed that differences measured with the pressure platform were all inferior to the standard error obtained with the force platform for every intersegmental moment (*p* < 0.001).

**Interpretation:**

The use of a simulated pressure platform to determine intersegmental moments has the same clinical efficiency as force platforms. Moreover, the possibility to set the platform into the radiograph room will allow in a second time a correlation between radiographic parameters and biomechanical constraints applied to the spine.

## 1. Introduction

Importance of spinal global sagittal alignment has been widely described in the literature and correlated with clinical outcomes [1–3]. Analysis of these radiographic parameters has led to a better understanding of normal alignment [4] as well as age-related changes [5, 6]. Furthermore, integration of pelvic parameters in spinal assessment was highly correlated with clinical outcomes [7] and helped to understand which factors were related to spinal deformities and which were acting as compensatory mechanism in order to maintain the head over the pelvis and a horizontal gaze [7–10]. The initial change in adult spinal deformity is a loss of lumbar lordosis, leading to an anterior global malalignment, which can potentially be worsen by an increase of thoracic kyphosis. In order to compensate this “anterior fall” of the trunk, pelvis will progressively rotate backward (retroversion) and finally knees will flex in order to maintain body weight over the feet. At the same time, this global malalignment will be associated with an increased cervical lordosis to maintain horizontal gaze [11].

However, one of the limitations of these studies is related to the fact that they are highly dependent on the quality of the spinal radiographs, and it has been shown that this “radiographic posture” does not always represent real alignment of the patient [12], especially in case of significant anterior malalignment. Recent development of the three-dimensional imaging system has improved our ability to assess spinal alignment [13], and significant differences have been revealed between two-dimensional and three-dimensional analyses using a force platform [14, 15]. Standing posture might therefore not be considered as a static condition but more like a permanent control of equilibrium.

On the contrary, various studies have analyzed posture and balance strategies using center of pressure volunteers, patients with chronic low back pain or postoperatively [16–19]. Recently, a new postural analysis protocol was described calculating moments applied on different body segments using a force platform and skin markers [20]. However, this approach is done in a totally free standing position, and it requires a movement analysis laboratory and can be difficult to generalize to clinical centers. Therefore, it may be interesting to adapt this biomechanical postural approach to a more clinically applicable form, using data from full-spine radiographs and a pressure platform. The aim of this study was therefore to evaluate with this protocol the differences between the uses of a pressure platform vs. a force platform. In other words, if we consider the standing posture as a nonstatic condition, can we neglect the horizontal ground reaction force?

## 2. Methods

### 2.1. Study Sample

Postural data from two healthy adult volunteers were analyzed for this experimental study.

### 2.2. Postural Analysis Protocol and Data Acquisitions

Analysis of the posture of each volunteer was done according to a previously described protocol [20] providing sagittal intersegmental moments and summarized hereafter. A set of 36 markers was used to divide the body in 10 segments (head, thorax, abdomen, pelvis, thighs, legs, and feet), and then a mass was attributed to each segment according to Dumas et al. [21] using anthropometric tables and the height/weight of the subject.

Once equipped, the two volunteers were asked to adopt 4 times a free-standing position without external constraint or support, which each foot positioned over a force plate (AMTI, Watertown, MA, USA) in order to collect the ground reaction force of the subject. During acquisition, the location of the markers over time were recorded using a Vicon® (Vicon, Oxford, UK) optoelectronic system with 6 high-resolution infrared cameras and a 100 Hz sampling frequency. Analysis of the acquired data was conducted on a one-second record sample, with the less body sway, in order to calculate mean intersegmental moments in the sagittal plane at each joint center.

Postacquisition calculations were done in order to obtain the sagittal net intersegmental moments (ankles, knees, hips, lumbo-sacral junction, thoraco-lumbar junction, and cervico-thoracic junction) using an ascending manner (i.e., going upward from the ground reaction forces) between each body segment previously identified except for the cervico-thoracic junction, where the sagittal net articular moment was calculated using a descending manner from the center of mass and mass of the head and neck.

### 2.3. Comparison between Intersegmental Moments and Statistical Analysis

Experimental error measurement of the protocol was done in a previous work according to Schwartz et al. [22] methodology and allowed to obtain intrasubject, intraobserver, and interobserver variability of the protocol. These results were obtained using a force plate giving three-dimensional ground forces and moments (*x* = anterior-posterior, *y* = vertical, and *z* = lateral).

In order to evaluate the differences in terms of intersegmental moments between measurements done using a force or a pressure platform, the postacquisition calculations were done with a simulated pressure platform. Only the vertical component (*y*) was kept in order to simulate the pressure platform, and results of these “pressure” intersegmental moments were compared to the “force” moments using a *t*-test with a level of significance set at 5%. Finally, the mean difference between “force” and “pressure” intersegmental moments were compared to the experimental error measurement obtained with the original protocol.

In this study and according to the high correlations between sagittal alignment and clinical outcomes, only sagittal intersegmental moments were analyzed (moments *z*).

## 3. Results

### 3.1. Study Sample

The first volunteer was a 30-year-old male, (180 cm, 80 kg) and the second volunteer was a 26-year-old female (158 cm, 52 kg).

### 3.2. Comparison between Force vs. Pressure Platform in terms of Mean Intersegmental Moments

After calculation, mean segmental moments (“pressure” and “force”) between each body segment for each volunteer were compared. Results from this comparison (Table 1) showed significant differences between the 2 platform configurations in terms of intersegmental moments for the lumbo-sacral junction, hips, knees, and ankles (*p* < 0.005). Significant difference was found neither for the thoraco-lumbar junction (*p*=0.162) nor for the cervico-thoracic junction but due to the fact that this moment was calculated in a descending manner without taking into account ground reaction force.

### 3.3. Comparison between Force vs. Pressure Platform in terms of Experimental Error Measurement

Differences in terms of intersegmental moments (in absolute values) were used in order to compare experimental error measurements with the simulated pressure platform with the previous experimental error measurements obtained with the force platform. Intrasubject comparison revealed that only the variability of hip moments measured with the pressure platform was superior (*p*=0.213) to the standard error calculated with the force platform, while variability of lumbosacral, knee, and ankle moments calculated with the simulated platform were all inferior to intrasubject variability measured with the force platform (*p* < 0.001).

Results from intra- and interobserver comparisons showed that differences measured with the pressure platform were all inferior to the standard error obtained with the force platform for every intersegmental moment (*p* < 0.001).

## 4. Discussion

While postural analysis can be done using various protocols, evaluation of intersegmental moments is, to date, a new way to express posture in terms of clinical efforts needed to maintain a stable balance. In order to develop the use of this protocol in clinical practice, a combined analysis with full-spine radiographs and a pressure platform can be a valuable alternative. The aim of this study was to evaluate the comparability of the pressure platform vs. force platform using the same protocol as we described previously. This protocol has shown its accuracy for the calculation of intersegmental moments [20].

To the best of our knowledge, this is the first study describing the use of a simulated pressure platform to calculate the intersegmental moments. According to the findings of the present work, using a pressure platform instead of a force platform leads to significant differences on various intersegmental moments. However, the mean differences between the values obtained with the pressure and the force platforms were almost all significantly inferior to the mean experimental measurement error. In other words, the differences observed between the 2 different ways of measurement are not clinically significant, and horizontal ground reaction force can be neglected for intersegmental moment measurements during the standing posture. Thus, using pressure platforms for the evaluation of the intersegmental moments will represent a valuable alternative to the use of force platforms in daily practice.

A growing number of studies underline the importance of analyzing the center of pressure in patients with spine disorders. Brumagne et al. have shown that patients with chronic low back pain, in addition with changes in postural adaptation, had a significant modification of the position of their center of pressure, in comparison with healthy subjects [23]. Moreover, this kind of data provides information about the constraints that apply at different levels of the spine. Various authors have described the links that exist between low back pain and postural changes of the spine [17, 18, 24]. Analyzing intersegmental moments in these patients could be of great interest. Recently, Bailey et al. [25] reported the results of a biomechanical study that evaluated peak sagittal vertical axis, forces, and muscular moments at various joints. According to their results, a significant improvement of dynamic sagittal balance metrics was observed after surgical correction in the setting of adult spinal deformity.

The advantage of using a pressure platform instead of a force platform is also related to the fact that this device is portative and can be set up in a radiograph room. By this way, correlations between intersegmental moments and radiographic parameters could be analyzed, and correlation with postural adaptation of the spine could be revealed using a simple method in daily practice. Further steps are still needed in order to use these results in daily practice. Among them, we are currently investigating the differences between intersegmental moments using skin markers and simulated markers placed directly on the X-rays. If validated, this point will then allow us to get rid of skin markers and try to correlate intersegmental moments with radiographic parameters.

## 5. Conclusions

The understanding of postural adaptation of the spine consecutive to degenerative changes is a great issue for the management of age-related spinal disorders. The use of a pressure platform to determine intersegmental moments might have the same clinical efficiency as force platforms. Moreover, these findings may open new possibilities in standing posture evaluation such as the use of a pressure platform into the radiograph room in order to correlate radiographic parameters and biomechanical constraints applied to the spine.

## Figures and Tables

**Table 1:** Mean difference (in absolute values) in terms of intersegmental moments between force and pressure platforms (in N·m).

	T-L	L-S	LHip	RHip	LKnee	RKnee	LAnkle	RAnkle
Mean difference	0.161	0.191	1.562	1.774	0.836	1.096	0.062	0.365
*p* value	0.162	0.048	<0.001	<0.001	<0.001	<0.001	0.006	<0.001

T-L = thoraco-lumbar junction; L-S = lumbo-sacral junction; L/RHip = left/right hip; L/RKnee = left/right knee; L/RAnkle = left/right ankle.

## Data Availability

Data related to the study are available on request.
